# Association of Adherent-invasive *Escherichia coli* with severe Gut Mucosal dysbiosis in Hong Kong Chinese population with Crohn’s disease

**DOI:** 10.1080/19490976.2021.1994833

**Published:** 2021-11-23

**Authors:** Zhilu Xu, Xiangqian Dong, Keli Yang, Caroline Chevarin, Jingwan Zhang, Yu Lin, Tao Zuo, Lok Cheung Chu, Yang Sun, Fengrui Zhang, Francis Kl Chan, Joseph Jy Sung, Jun Yu, Anthony Buisson, Nicolas Barnich, Jean-Frédéric Colombel, Sunny Hei Wong, Yinglei Miao, Siew C Ng

**Affiliations:** a Department of Medicine and Therapeutics, Institute of Digestive Disease, State Key Laboratory of Digestive Diseases, Lks Institute of Health Science, the Chinese University of Hong Kong, Hong Kong, China; b Center for Gut Microbiota Research, Faculty of Medicine, The Chinese University of Hong Kong, Hong Kong, China; c Department of Gastroenterology, The First Affiliated Hospital of Kunming Medical University, Yunnan, China; d Yunnan Province Clinical Research Center for Digestive Diseases, Kunming, Yunnan, China; e Centre De Recherche En Nutrition Humaine Auvergne, Université Clermont Auvergne, Inserm U1071, Usc-inrae 2018, Microbes, Intestin, Inflammation Et Susceptibilité De l’Hôte (M2ish), Clermont-Ferrand, France; f 3iHP, Chu Clermont-Ferrand, Service d’Hépato-Gastro Entérologie, Clermont-Ferrand, France; g Department of Gastroenterology, Icahn School of Medicine, Mount Sinai, New York, USA

**Keywords:** Adherent-invasive Escherichia coli, AIEC, Crohn’s disease, Inflammatory bowel disease, Microbiota

## Abstract

Adherent invasive *Escherichia Coli* (AIEC) has been implicated in the pathogenesis of Crohn’s disease (CD) in Western populations. Whether the presence of AIEC is also seen in CD populations of different genetic susceptibility and has negative impact on host microbiota ecology and therapeutics are unclear. AIEC presence was assessed in ileal tissues of 60 Hong Kong Chinese patients with CD and 56 healthy subjects. Mucosa microbiota was analyzed by 16s rRNA sequencing. Impact of AIEC on the gut microbiota was determined in a mouse model. AIEC was significantly more prevalent in ileal tissues of patients with CD than controls (30% vs 7.1%). Presence of AIEC in ileal tissues was associated with more severe mucosa microbiota dysbiosis in CD with decreased diversity and lower abundance of Firmicutes including butyrate producing *Roseburia* and probiotic *Bacillus*. A random forest model predicted the presence of AIEC with area under the curve of 0.89. AIEC exacerbated dysbiosis in dextran sodium sulfate (DSS)-induced colitis mice and led to resistance to restoration of normal gut microbiota by fecal microbiota transplantation (FMT). Proportion of donor-derived bacteria in AIEC-colonized mice was significantly lower than that in uninfected mice. AIEC was prevalent and associated with severe mucosa microbiota dysbiosis in CD in Hong Kong Chinese population. The presence of AIEC impeded restoration of normal gut microbiota. AIEC may serve as a keystone bacterium in CD and impact the efficacy of FMT.

## Introduction

Crohn’s disease (CD) is one of the major types of inflammatory bowel disease (IBD). It might result from a complex interplay between genetic susceptibility, environmental factors, and disrupted gut microbiota.^1^ The incidence of CD is increasing globally especially in newly industrialized countries.^2^ Changes in composition of the intestinal bacterial community and reduced microbiota richness and diversity have been observed in CD.^3^ Importantly, adherent-invasive *Escherichia coli* (AIEC) have been frequently recovered from mucosa of CD patients in areas with high CD incidence,^4–6^ where diet is predominantly high in fat and sugar content, which may increase the host’s susceptibility to AIEC infection.^7^ Host polymorphisms in autophagy-related genes such as domain-containing protein 2 (NOD2) could also lead to impaired ability to resist AIEC colonization.^8^ However, these genetic and environmental factors favoring AIEC colonization are largely absent in the Chinese populations.^9^ It is unclear whether such putative pathobionts identified in Western populations are present in IBD patients in regions with increasing IBD incidence, such as Hong Kong where the incidence of CD has increased by 30-fold in the past two decades.^2^

AIEC bacteria play a central role in promotion of inflammation or perturbation of immune homeostasis within and beyond the intestine.^6,10,11^ They could initiate inflammatory responses and induce microbiota dysbiosis in immune deficient mice,^11–13^ which might play a driver role in gut inflammatory diseases.^3^ However, to date, no investigation has addressed the impact of AIEC on gut microbiota in humans. Here, we investigated the prevalence of AIEC in a Hong Kong Chinese population, delineated the mucosal microbiota of CD patients in association with the presence of AIEC and the impact of AIEC on gut microbiota restoration via fecal microbiota transplantation (FMT), a potential therapeutic strategy that could restore the appropriate host-microbiota crosstalk, in an animal model.

## Results

### Prevalence and characteristics of AIEC in Hong Kong Chinese population

We examined the presence of AIEC in 56 healthy controls and 60 patients with CD, including 22 patients with inflamed terminal ileum and 38 patients with noninflamed terminal ileum. Paired tissues from inflamed and noninflamed areas were taken from five patients with partial ileal inflammation (Table 1 and 2 **and** supplementary Table 1). AIEC was present in the ileal mucosa of 18 (30%) of 60 patients with CD and 4 (7.1%) of 56 healthy controls (*p* < .05, Chi-squared test). The presence rate of AIEC in tissues from inflamed and noninflamed area was comparable (31.8% and 28.9%). For paired tissues collected from patients with partial inflamed ileum, AIEC strains were recovered from either both (n = 2), or none (n = 3) of the inflamed and noninflamed tissues. The mucosal-associated *E. coli* load in inflamed tissues showed no significant difference compared noninflamed ileal tissues (*p* = .49, supplementary Figure 1a-b). The invasion abilities of AIEC strains isolated from inflamed tissues showed no significant difference compared with AIEC strains isolated noninflamed ileal tissues (*p* = .81, supplementary Figure 1c). Twelve (57.1%) of 21 of CD mucosa-associated AIEC strains were gentamicin resistant. Based on the presence of AIEC in mucosal tissues, the CD patient samples were grouped as AIEC-positive and AIEC-negative. Mucosal-associated *E. coli* load in AIEC-positive tissues was higher than AIEC-negative tissues (*p* = .064 for CD tissues and *p* < .001 for control tissues, *t* test, supplementary Figure 1d).

**Table 1.** Demographic characteristics of the cohort

	CD (n = 60)			
Group	AIEC positive	AIEC negative	P value *	HC (n = 56)
Age, mean ± SD	50 ± 15.1	43.9 ± 15.3	0.145	53.2 ± 10.3
Gender, female, n (%)	9 (50%)	14 (33%)	0.257	32 (57.1%)
Current/ex. smoker, n (%)	5 (28%)	5 (12%)	0.149	
Condition, inflamed, n (%)	7 (39%)	15 (36%)	1.000	
Disease activity, HBI, n (%)				
mild	17	37	0.658	
moderate	1	3	1.000	
severe	0	2	1.000	
Medication, n (%)				
Topical treatment	1 (5.6%)	0	0.300	
Systematic ASA	8 (44%)	13 (31%)	0.381	
Systematic steroids	2 (11%)	1 (2.4%)	0.212	
Immunosuppressants	14 (78%)	26 (62%)	0.371	
Anti-TNF	8 (44%)	10 (24%)	0.133	
Antibiotics	0	0		
Extraintestinal manifestations, n (%)	0	0		

* Fisher’s exact test was used for statistical comparison between categorical data. Unpaired *t* test was used for statistical comparison between numerical data.

**Figure 1.**
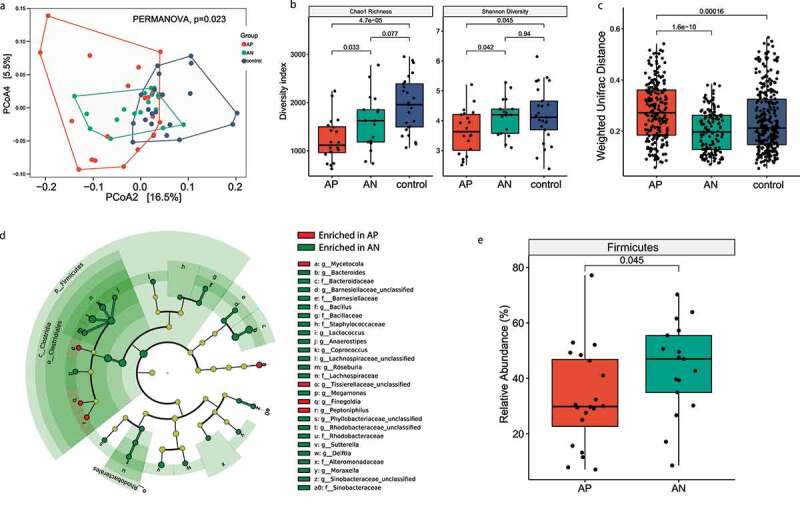
Mucosal microbiota in healthy subjects and CD patients with or without the presence of AIEC. (A) Microbiota Chao1 richness and Shannon diversity index in mucosal samples were depicted, significance was calculated based on Wilcoxon rank-sum test; (B) PCoA analysis of mucosal samples based on weighted UniFrac distance matrix was depicted. Significant difference in microbiome composition was observed among AP, AN, and control tissues (*p* = .023, PERMANOVA). Subgroup analysis showed that the microbiome composition in AIEC-positive CD tissues was significantly different compared to control tissues (*p* = .048, PERMANOVA); (C) intragroup weighted UniFrac distances of AP tissues was significantly higher than AN and control tissues (*p* < .001, Wilcoxon rank-sum test); (D) cladogram depicting differentially abundant taxa between AP and AN tissues by LEfSe. Taxa with LDA>2 and adjusted *p* < .05 were shown. (E) Relative abundance (%) of Firmicutes phylum was depicted. AP, AIEC positive; AN, AIEC negative; PCoA, principal coordinate analysis. LEfSe: Linear discriminant analysis Effect Size; LDA: Linear discriminant analysis

**Table 2.** Characteristics of AIEC strains isolated in Hong Kong Chinese population

STRAIN	Invasion rate compared to initial inoculum (%)	Adhesion rate compared to initial inoculum (%)	Source of strain
1162d	4.25%	225.00%	Crohn’s Disease
1177c	2.23%	90.00%	Crohn’s Disease
1194b	3.35%	155.00%	Crohn’s Disease
1133a	0.91%	200.00%	Crohn’s Disease
1186IFc	2.03%	165.00%	Crohn’s Disease
1186NIFa	2.35%	240.00%	Crohn’s Disease
1100a	2.48%	190.00%	Crohn’s Disease
1105d	0.80%	36.50%	Crohn’s Disease
1010a	0.43%	52.00%	Crohn’s Disease
1099a	0.95%	45.00%	Crohn’s Disease
1111a	0.83%	175.00%	Crohn’s Disease
1200c	0.53%	107.50%	Crohn’s Disease
1218a	1.38%	99.00%	Crohn’s Disease
1219a	0.66%	21.00%	Crohn’s Disease
1221a	0.56%	13.75%	Crohn’s Disease
1222a	1.22%	140.00%	Crohn’s Disease
1222b	0.57%	60.00%	Crohn’s Disease
1272a	2.55%	90.00%	Crohn’s Disease
1273c	2.73%	160.00%	Crohn’s Disease
1282a	1.11%	50.00%	Crohn’s Disease
3003b	0.53%	100.80%	Non IBD
3013b	1.32%	12.50%	Non IBD
8223a	0.53%	100.80%	Non IBD
8226a	1.32%	12.50%	Non IBD
LF82	4.00%	120.00%	AIEC reference strain
K12	0.04%	0.50%	Nonpathogenic *E. coli*

### AIEC is associated with dysbiosis in patients with CD

To investigate whether the presence of AIEC impacted the human mucosa microbiota, 16s ribosomal RNA sequencing was performed on 63 biopsy samples, including 21 biopsies from 18 AIEC positive CD patients (3 pairs of inflamed and noninflamed tissues), 17 biopsies from AIEC negative patients and 25 biopsies from healthy controls. The Chao1 richness in CD tissues was significantly lower than control tissues (*p* < .001, Wilcoxon rank-sum test), whereas no significant difference in Shannon diversity index was observed between CD and control tissues (*p* = .19, Wilcoxon rank-sum test, Supplementary Figure 1e). The overall microbiome composition in CD tissues was significantly different compared to control tissues (*p* = .019, PERMANOVA, Figure 1a). The Shannon diversity index and Chao1 richness in AIEC-positive tissues was significantly lower than AIEC-negative and control tissues (*p* = .033 and *p* = .042, respectively, Wilcoxon rank-sum test, Figure 1b). Significant difference in microbiome composition was observed among AIEC-positive CD tissues, AIEC-negative CD tissues and control tissues (*p* = .023, PERMANOVA, Figure 1a). Subgroup analysis showed that the microbiome composition in AIEC-positive CD tissues was significantly different compared to control tissues (*p* = .048, PERMANOVA, Figure 1a). No significant difference was observed between AIEC-negative tissues and control tissues (*p* = .052), as well as between AIEC-positive and AIEC-negative CD tissues (*p* = .249, PERMANOVA, Figure 1a). The intragroup variation of AIEC-positive microbiota was significantly higher than AIEC-negative tissues (*p* < .001, Wilcoxon rank sum test, Figure 1c). Linear discriminant analysis effect size (LEfSe) analysis revealed 76 taxa that were differentially abundant between AIEC-positive and AIEC-negative microbiota (LDA>2, *p* < .05, Figure 1d). AIEC-positive tissues were enriched with *Peptoniphilus, Finegoldia* and *Mycetocola* and depleted with *Bacteroides, Coprococcus, Sutterella, Roseburia, Megamonas, Delftia, Moraxella, Bacillus, Lactococcus, Anaerostipes*, compared with AIEC-negative tissues (LDA>2, *p* < .05, Figure 1d). At the phylum level, AIEC-positive tissues were depleted with Firmicutes compared with AIEC-negative tissues (*p* = .045. Wilcoxon rank-sum test, Figure 1e).

### Mucosa microbiota composition was predictive of AIEC presence in patients with CD

**Figure 2.**
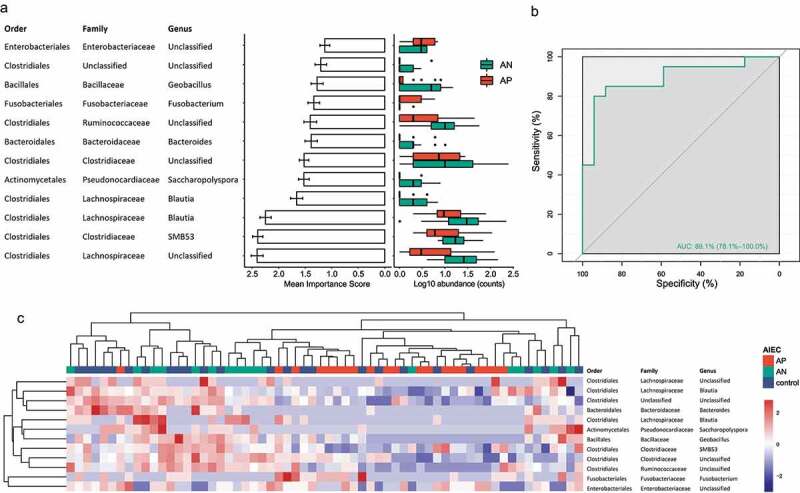
Classification for the presence of AIEC based on mucosal microbiome signature through a RF model. AP and AN samples were divided into 80% of training set (n = 30) and 20% test set (n = 7) by randomized stratified sampling. Importance of each feature was determined by the training set and the prediction accuracy of the model was determined by the test set. (A) Taxonomy, importance, and relative abundance of the 12 markers with highest average importance score for the final model in AP and AN. The abundances of the markers were plotted on a logarithmic scale, and values of zero are assigned to count = 1. (B) The RF prediction model with area under ROC of 0.89 for the test set. (C) Hierarchical clustering based on abundance of 12 marker OTUs that were discriminative for AIEC positive and AIEC negative CD tissues. AP, AIEC positive; AN, AIEC negative; CD: Crohn’s Disease; RF, random forest; ROC, receiving operational curve

We next assessed the potential value of using gut microbiota as biomarkers for predicting the presence of AIEC and found that a random forest model based on 12 markers yielded an area under the receiver operating characteristic (AUC) of 0.89 (Figure 2a-b, supplementary Figure 2). The model showed AIEC-negative average of 0.83 in sensitivity, 0.85 in specificity, and 0.84 overall accuracy for the test set. Among 12 markers, two belonging to Proteobacteria and Fusobacteria were enriched in AIEC-positive samples, while 10 belonging to Actinobacteria, Bacteroidetes or Firmicutes were depleted in AIEC positive samples (Figure 2b). Hierarchical clustering based on abundance of these 12 markers showed that most AIEC positive tissues formed a distinct cluster from AIEC negative tissues and control tissues (Figure 2c), suggesting that these taxonomic changes were specific to the presence of AIEC.

### Microbiota functional dysbiosis in AIEC-positive tissues

**Figure 3.**
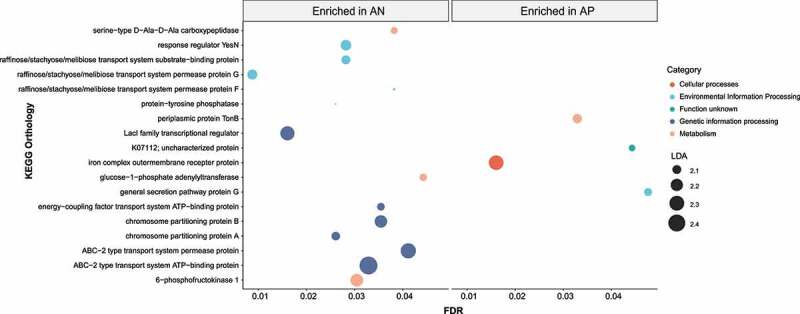
Functional analysis of predicted metagenomes. Bubble plot depicting the differentially abundant KEGG Orthologs between AP (n = 20) and AN (n = 17) identified by LEfSe. The x axis represents adjusted *p* values while size of the bubble plot represents the LDA effect size of each KO. AP, AIEC positive; AN, AIEC negative; LDA, linear discriminant analysis; LEfSe, linear discriminant analysis effect size

To study the functional and metabolic changes of the microbiome communities between AIEC-positive and AIEC-negative tissues, we next inferred the functional potential from the 16S rRNA data using PICRUST2. LEfSe analysis identified 18 KEGG Orthology (KO) that were significantly different between AIEC-positive and AIEC-negative CD tissues (LDA >2.0, *p* < .05, Figure 3). Multiple KOs within the categories of genetic information processing and environmental information processing were depleted in AIEC-positive tissues, compared with AIEC-negative tissues. Metabolic Pathways related to Lipopolysaccharide (LPS) biosynthesis (NAGLIPASYN-PWY and PWY-6467) were significantly enriched in AIEC-positive tissues while pathways of several amino acid biosynthesis (L-arginine, L-lysine, L-isoleucine etc.) were depleted in AIEC-positive tissues, compared with AIEC-negative tissues (supplementary Table 2).

### AIEC exacerbated microbiota dysbiosis in DSS-induced colitis mice and hindered the restoration of normal gut flora by FMT

**Figure 4.**
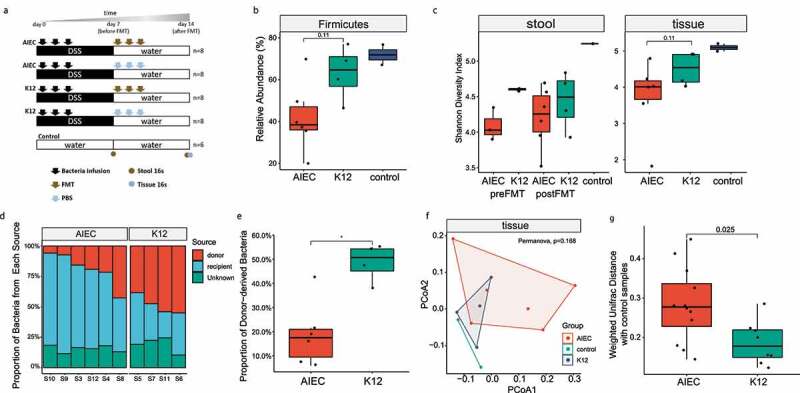
The impact of AIEC on gut microbiota in mice following FMT. (A) Study scheme; (B) Boxplot depicting the relative abundance (%) of Firmicutes phylum in the mucosal microbiota of AIEC mice and K12 mice; (C) The Shannon diversity index in fecal and mucosal microbiota in AIEC mice and K12 mice; (D–E) Proportion of recipient mice microbiota derived from difference sources determined by SourceTracker. “Unknown” refers to the OTUs in the recipient microbiota after FMT that was not derived from either the donor or the recipient mice before FMT; (F) PCoA analysis based on weighted UniFrac matrix of mucosal microbiota in AIEC mice, K12 mice and control mice. (G) Boxplot depicting the weighted UniFrac distance between post FMT samples in AIEC mice and the control mice, and K12 mice and the control mice, respectively. PCoA, principal coordinate analysis Difference between groups were analyzed by Wilcoxon rank sum test. * *p* < .05

We further elucidated the impact of AIEC on gut microbiota in a dextran sodium sulfate (DSS) induced colitis mouse model. C57BL/6 mice were inoculated with an AIEC strain isolated from Hong Kong CD patients (AIEC 62d), or a nonpathogenic *E. coli* strain K12 (Figure 4a). The AIEC strain was chosen due to its comparable invasion ability as the AIEC reference strain LF82. Feces from healthy mice were infused into AIEC infected mice to study whether AIEC-associated dysbiosis could be restored via FMT (Figure 4a). In accordance with human data, AIEC mice harbored lower Firmicutes in colonic mucosa associated microbiota compared with K12 mice (*p* = .11, Wilcoxon rank-sum test, Figure 4b). The bacterial genera *Allobaculum, Anoxybacillus, Sutterella, Selenomonas, Bifidobacterium, Adlercreutzia, AF12*, and *Anaerofustis* were significantly depleted in AIEC mice compared to K12 mice (LDA>2, *p* < .05, supplementary Figure 3). The Shannon diversity index in mucosa of AIEC-infected mice was lower than K12 mice (*p* = .11, Wilcoxon rank-sum test, Figure 4c). An average of 50.1% of microbes in K12 mice after FMT originated from donor, while the proportion of donor-derived microbes constitute only 17.5% in AIEC mice (*p* < .05, Wilcoxon rank-sum test, Figure 4d-e). The weighted UniFrac distance between tissue samples from K12 mice and control mice were significantly smaller than that between AIEC-infected mice and control mice (*p* = .025, Wilcoxon rank sum test, figure 4f-g), indicating that the tissue microbiota of K12 mice resembled more to that of the control mice, compared to AIEC-infected mice.

### Persistent AIEC infection hindered the recovery of colitis

**Figure 5.**
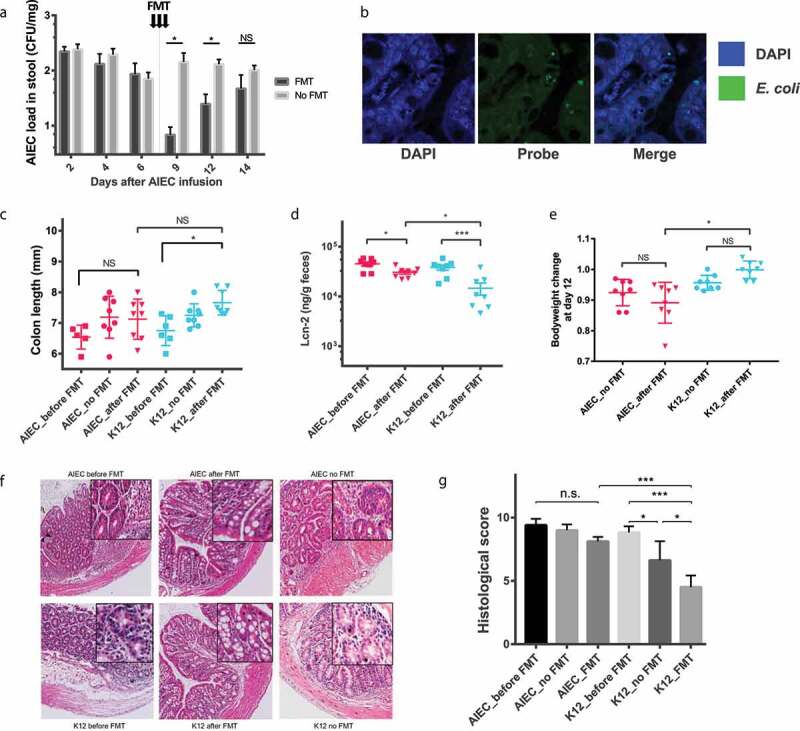
The impact of AIEC infection on the recovery of colitis following FMT.(A) AIEC load in mice stool represented by log10 value of CFU/mg faces; (B) FISH staining showing the location of intracellular *E coli*. (Blue: DAPI, Green: E. coli specific probe); (C) colon length (mm) of mice at sacrifice; (D) Fecal Lcn-2 level before and after FMT; (E) Change of bodyweight at day 12 of the experiment (3 days since last FMT); (F–G) HE staining of mice colon and histological score of AIEC and K12 mice before and after FMT. FISH, Fluorescence *in situ* hybridization; * *p* < .05, ** *p* < .01, *** *p* < .001

Fecal AIEC level in mice receiving FMT was significantly lower than that in mice receiving PBS control at day 9 and 12 (0 ~ 3 days post last FMT, *p* < .05, Wilcoxon rank sum test, Figure 5a). However, the fecal AIEC level was not significant different between mice receiving FMT and PBS at day 14 (Figure 5a). Fluorescence in situ hybridization (FISH) showed remaining AIEC within the epithelial cells of mice colon after FMT treatment (Figure 5b). After FMT, the colon length of K12-inoculated mice, but not AIEC-infected mice, was significant longer than that before FMT (*p* < .05, Tukey’s test, Figure 5c). The post FMT fecal Lcn-2 level at day 14 in both in K12-inoculated mice and AIEC-infected mice was significantly lower than that before FMT (*p* < .001 and *p* = .048, Tukey’s test, Figure 5d). The post FMT fecal Lcn-2 level in AIEC-infected mice was significantly higher compared to K12 mice at day 14 (*p* = .028, Tukey’s test, Figure 5d). At day 12, the bodyweight of K12 mice after FMT was significantly higher compared AIEC-infected mice after FMT (*p* < .001, Tukey’s test, Figure 5e). The histological score in K12 mice receiving FMT was significantly lower than K12 mice receiving PBS and before FMT (*p* = .012 and *p* < .001, Tukey’s test, Figure 5f-g). The post FMT histological score of AIEC-infected mice was significantly higher compared to K12 mice (*p* < .001, Tukey’s test, Figure 5f-g). AIEC-infected mice, on the other hand, showed no significant improvement of histological score after FMT, compared with AIEC-infected mice receiving PBS and before FMT (Figure 5f-g).

## Discussion

We report that the prevalence AIEC in Hong Kong Chinese population was comparable to that in western counties, suggesting that these bacteria may be a risk factor globally despite different genetic background and lifestyle between Asian and Caucasians. We showed, for the first time, that AIEC was associated with mucosa microbiota dysbiosis in humans. Using an animal model, we confirmed that AIEC infection was associated with decrease of microbiota diversity and abundance of Firmicutes, mimicking the microbiota dysbiosis pattern in humans. AIEC could hinder the restoration of normal gut microbiota by FMT and stultify the recovery of chemical induced colitis, highlighting the role of AIEC in initiating and sustaining mucosa microbiota dysbiosis.

In CD patients with partial inflamed terminal ileum, AIEC strains were recovered from either both, or none of the inflamed and noninflamed tissues, suggesting that AIEC colonize the ileal in an all-or-none manner, and that AIEC may not require preexisting inflammation to colonize the human gut. This indicates that the presence of AIEC is not a consequence of the inflammation but may play a causal or aggravating role of the disease, reinforcing the efforts that must be made to limit these bacteria in CD patients.

Currently, the identification of AIEC strains purely relies on the assessment of the interaction between bacteria and host cells. This culture-based method is highly time and labor consuming and requires strict handling of clinical samples to retain the viability of bacteria. Lack of specific molecular marker for distinguishing AIEC pathovar from other *E. coli* strains^14–16^ made it impossible to study the impact of AIEC on microbiota in previous microbiome studies. By collecting multiple ileal biopsies from same patients and assessing the presence of AIEC and mucosa microbiota in parallel, we were able to stratify our CD cohort into AIEC-positive and AIEC-negative groups. A random forest model composed of 12 markers was able to accurately distinguish AIEC-positive and AIEC-negative microbiota. Although this was a single center study with limited sample size, we provide an alternative way for AIEC detection based on mucosa microbiome profiles.

The mucosa microbiota in AIEC-positive patients showed more severe dysbiosis with significant lower bacterial diversity compared with AIEC-negative and control microbiota. The mucosal microbiota in mice colonized with AIEC also showed lower diversity compared with uninfected mice. This indicates that AIEC infection was associated lower richness and evenness of the mucosal microbiota. In humans, AIEC-positive microbiota had lower abundance of several genera including *Coprococcus, Roseburia, Bacillus, Lactococcus*, and *Anaerostipes* compared with AIEC-negative microbiota. These genera were all reported to be depleted in IBD patients compared with healthy controls.^3,17–19^ In mice colonized with AIEC, the mucosal microbiota also showed depletion of beneficial genera such as Bifidobacterium, compared with K12 mice. Notably, both human and mice infected with AIEC showed significant depletion of the Firmicutes phylum, which were consistently reported to be associated with CD.^3,17,20,21^ The resemblance of AIEC associated dysbiosis in both human and mice toward CD-like microbiota indicates that AIEC might play a causal role in instigating dysbiosis by promoting mucin degradation and penetrating the mucous layer, thus enhance the translocation of bacteria through the mucous layer and adhesion to intestinal epithelial cells.^22–24^

The mucosa microbiota in AIEC-positive patients was enriched with LPS biosynthesis and LPS biosynthesis proteins, which lead to increase of proinflammatory cytokines.^25^ This is in line with previous study that AIEC colonization drove chronic inflammation, which correlated with microbiota components having higher levels of bioactive LPS and flagellin in Toll-like receptor 5 knock out mice.^11^ In addition, pathways of several amino acid biosynthesis (L-arginine, L-lysine, L-isoleucine etc.) were depleted in AIEC-positive tissues, compared with AIEC-negative tissues. These results suggest that AIEC-infection was associated with functional dysbiosis of the mucosal microbiota.

FMT was effective in ameliorating DSS-induced experimentally colitis and initiating the restoration of intestinal homeostasis.^26^ Conceptually, the restoration of a ‘healthy’ intestinal microbiota could reverse the inappropriate immune mucosal stimulation in CD and create a less suitable environment for AIEC colonization. However, our data showed that FMT only transiently reduced fecal AIEC load but was not able to completely eradicate AIEC from the mouse gut in DSS colitis model. AIEC infection led to impaired efficacy of FMT on colitis and hindered the restoration of a ‘healthy’ microbiota. The presence of AIEC was significantly associated with lower proportion of donor-derived microbes following FMT, which is associated with poor treatment outcome.^27^ The efficacy of FMT treatment varies across recipients with difference microbiome profile. AIEC can induce increased expression of TNF-α, IFN-γ and IL-8, leading to recruitment of macrophages and dendritic cells to the site of infection.^6^ The increased immune response may make it harder for commensal and beneficial bacteria to colonize in the inflamed gut. Moreover, AIEC can also cause intestinal barrier dysfunction (leaky gut), making it more vulnerable for other bacteria to penetrate the gut barrier. Therefore, by introducing a variety of foreign bacteria into the inflamed gut, FMT may not improve, if not worsening, the severity of the disease.^28^ These data suggest that AIEC impacts FMT efficacy by hindering engraftment of donor-derived bacteria, leading to incomplete recovery of intestinal inflammation and restoration of normal gut flora.

In conclusion, AIEC is associated with ileal CD in Hong Kong Chinese population. Presence of AIEC is associated with severe mucosal microbiota dysbiosis in CD patients and colitis mouse model. The presence of AIEC could compromise the efficacy of FMT by hindering the engraftment of beneficial bacteria. These data further suggest that AIEC positive ileal CD could represent a subtype of the disease in which specific eradication strategies should be considered.

## Material and methods

### Study population

Biopsies were obtained from 116 Hong Kong Chinese subjects (60 CD and 56 healthy controls) undergoing colonoscopy at the Prince of Wales Hospital, the Chinese University of Hong Kong. Patients were included if they were 18 years or older with a diagnosis of ileal CD defined by endoscopy, radiology, and histology (ileal or ileocolonic); had active ileal disease at the time of endoscopy and on stable CD-related medication. Subjects with a use of antibiotics, probiotics or prebiotics or a history of enteric infection in the past three months were excluded. Subjects with no prior gastrointestinal disease and a normal colonoscopy as part of screening checkup were recruited as healthy controls. Informed consent was signed by all participants. During colonoscopy, mucosal biopsies were taken from terminal ileum. Tissues were immersed in the cell culture medium MEM supplemented with 15% sterile glycerol, snapped frozen and stored at −80°C until use. This study was approved by the Ethics Committee of the Chinese University of Hong Kong (CRE 2014.026).

### AIEC isolation and characterization

Isolation and characterization of AIEC have been previously described.^14^ Briefly, the number of *E. coli* adhering to the intestinal mucosa were counted on Drigalski agar. Bacterial load was normalized by colony-forming units per milligram (CFU/mg) tissue samples. Colonies were cultured in 96-well microplates in Luria-Bertani medium supplemented with 15% glycerol and stored at −80°C. Adhesive and invasive properties were initially evaluated on intestinal epithelial cell line Intestine-407 (ATCC® CCL-6™) by antibiotic protection assay. Regarding the invasion, the results were expressed as percentage of the number of invasive bacteria compared to the number of bacteria present in the initial inoculum, defined as 100%. The survival and replication capabilities of invasive bacteria was measured within THP-1 macrophage (ATCC® TIB-202™). Survival rate was expressed as the mean number of bacteria recovered, compared with LF82, defined as 100%. *E. coli* strains were considered AIEC if the invasion rate was equal to or greater than 10% that of AIEC reference strain LF82.

### DNA extraction and 16S rRNA sequencing

For optimal isolation of bacterial DNA, mucosal biopsies were disrupted by bead-beating upon digestion in lysozyme (Sigma Aldrich, Northbrook, IL) before extraction and purification by Maxwell Promega Tissue DNA Purification kit according to manufacturer’s instructions. 16S ribosomal RNA gene amplicon sequencing was performed on DNA extracted from 63 biopsy samples (25 normal and 38 CD), including 21 biopsies from 18 AIEC positive patients (3 pairs of inflamed and noninflamed tissues) and 17 biopsies from AIEC negative patients. Mice fecal DNA was extracted using the QIAamp DNA Mini Kit according to manufacturer’s instructions. Mice colonic tissue DNA was extracted following same protocol as human tissues. The V3-V4 hypervariable region of 16S ribosomal RNA gene was amplified by forward primer 341 F-CCTAYGGGRBGCASCAG and reverse primer 806RGGACTACNNGGGTATCTAAT. Illumina HiSeq 2500 platform (Illumina, San Diego, CA) was used to generate PE 250 bp raw reads by Novogene (Beijing, China). PhiX was included for each sequencing run at a concentration of 15 nMol/ul (50% of the library).

### 16s rRNA analysis

Raw 16S rRNA amplicon reads were processed with FastX-Toolkit to remove reads with quality score below 20. Paired reads were then merged and filtered using VSEARCH^29^ remove reads with over 1% error rate, yielding an average of 65835 (11054–106171) (min-max) clean reads per sample (66813 (11054–106171) for human samples and 63910 (45479–80168) for mice samples). Remaining reads were converted to FASTA format and subjected to chimera filtering using VSEARCH de novo mode and the rdp_gold.fa database. Remaining reads were classified using the UTAX algorithm with RDP database and reads classified as either chloroplast or not classified at domain level were removed. Operational taxonomic units (OTU) tables were generated using the UPARSE algorithm^30^ implemented in VSEARCH software and all reads across samples. The remaining reads were then clustered at 97% similarity and annotated by the rdp_16s_v18.fa database. All reads per sample were then mapped to representative sequences using VSEARCH to generate OTU abundance tables. Human samples were rarefied to 12,126 reads One sample from AIEC-positive group with less than the said numbers were removed. Mice samples were rarefied according to the sample with lowest number of reads (45,479 reads per sample). The resulting OTU table and representative sequences were then used to predict the metabolic functional profiles with PICRUSt2 (Phylogenetic Investigation of Communities by Reconstruction of Unobserved States).^31^ Metabolic pathways were annotated using MetaCyc.^32^

### Multivariate machine learning analysis

Random forest (RF) was used to build AIEC-positive versus AIEC-negative prediction model using fecal microbes because of its superior performance for classification with binary features.^33^ AIEC-positive and AIEC-negative samples were divided into 80% of training set and 20% test set by random stratified sampling by caret R package. The importance value of each OTU to the classification model was evaluated by recursive feature elimination using the training set. Sequential forward selection was employed to identify most important OTUs. Model performances were compared in terms of binary classifiers with AUC of the test set. This process is repeated 100 times to avoid over fitting. OTUs with highest average importance scores were included in the model. The number of features was chosen when best accuracy for the test set was achieved. These analysis was done using R packages randomForest v4.6–14^34^ and pROC v1.15.3.^35^

### Animal model

The dextran sodium sulfate (DSS) colitis model was chosen due to its rapidity, simplicity, reproducibility, controllability, and compatibility for AIEC infection studies.^36,37^ Male C57B/L6 mice (7–8 weeks of age) were treated with 2% DSS for 7 days to induce colitis (Figure 4a). In a preliminary experiment, AIEC 62d was able to colonize the mice gut when mice were treated with a 7-day course of 2% DSS and persisted in the gut for at least 21 days after initial inoculation, with or without antibiotic treatment prior to gavage (supplementary Figure 4). Therefore, we gavage the mice with the *E. coli* strains without antibiotics pretreatment to minimize the disturbance of gut microbiota.

Mice were inoculated with 10^9^CFU of AIEC62d or nonpathogenic control strain K12 C600 for three consecutive days. Another group of mice were included as blank control and stool donors for FMT. At day 8, six mice from AIEC and K12 group were sacrificed as “before FMT” group. One mouse from AIEC group was moribund and euthanatized at day 5 and removed from subsequent analysis. Mice in FMT groups were treated with 200 μl of fecal solution (stool collected from control mice diluted 1:10 with sterile PBS) for three consecutive days. Mice in “no FMT” groups were treated with sterile PBS (n = 8 per group). Bodyweight of the mice was measured daily. Stool samples were collected every two days. DNA was extracted from stool and colonic tissues for 16S rRNA sequencing. For stool samples, pre-FMT and post-FMT samples were sequenced. For colonic tissue samples, post-FMT samples were sequenced (**supplementary table 3**). One stool sample from pooled FMT solution was sequenced as donor microbiome profile.

### Fecal bacterial load and assessment of inflammation

After weighing and serial dilutions, stool suspensions were plated on LB agar plates supplemented with 100 mg/L ampicillin and cultured at 37°C overnight to determine the AIEC load in mice gut (CFU/mg). Fecal lipocalin-2 (Lcn-2) were measured by ELISA according to manufacturer’s instruction (R&D). At sacrifice, colon length and histology score in distal colonic segments were measured to assess the severity of colitis. All slides were stained with hematoxylin and eosin (H&E). Blinded histologic scoring was performed. Each slide was assigned four scores by assessment of epithelial damage and inflammatory infiltration into the submucosa, mucosa, and muscularis/serosa, resulting in a total score of 12. Additional colon tissues from AIEC-infected mice were fixed in Carnoy’s solution for Fluorescence *in situ* hybridization (FISH) to visualize the location of *E. coli* within the mouse gut mucosa. Tissue sections were stained with 25 μg/mL FITC-labeled *E. coli* specific probe and nuclei was labeled with DAPI for confocal microscopy imaging (Leica confocal SP2 1P/FCS). High magnification images were obtained under the × 63 objective.

### Statistical analyses

All measurements were shown as mean ± standard deviation. Wilcoxon’s rank-sum test was used to test for differences in numerical variables, and Fisher’s exact test was used to evaluate the proportional difference in categorical variables between groups. Linear discriminant analysis Effect Size (LEfSe) analyses were performed on the Huttenhower lab Galaxy server (http://huttenhower.sph.harvard.edu/galaxy/) by importing the relative abundance values and associated sample metadata, with FDR-adjusted q value < 0.05 considered significant and effect size calculated. We used observed community richness and Chao1 community richness indexes for comparison of bacterial OTU richness and Shannon diversity indexes for comparison of bacterial OTU diversity. Origin of microbiota analysis was performed using SourceTracker v. 0.9.8 with default parameters.^38^ Donor and pre-FMT samples from same cage as source, and individual post-FMT samples as sink. Multiple group comparisons were made by the Kruskal Wallis test. *P* < .05 was considered statistically significant. All tests were performed with the R Project v3.6.0 for statistical computing or GraphPad Prism 7.0 (GraphPad, La Jolla, CA).

## Supplementary Material

Supplemental Material

## Data Availability

All sequence files are available from the SRA NCBI database under accession number PRJNA641238.

## Abbreviations

AIEC: Adherent-invasive Escherichia Coli; CD: Crohn’s disease; CFU: Colony-forming units; FMT: Fecal microbiota transplantation; OTU: Operational taxonomic units; CDAI: Crohn’s Disease Activity Index; RF: Random forest; AUC: Area under receiver operating characteristic curve; DSS: Dextran sodium sulfate; Lcn-2: Fecal lipocalin-2; FISH: Fluorescence in situ hybridization; PCoA: Principle coordinate analysis; LEfSe: Linear discriminant analysis Effect Size; LDA: Linear discriminant analysis; LPS: Lipopolysaccharide.
