# Supplementary material for: Association of Adherent-invasive Escherichia coli with severe Gut Mucosal dysbiosis in Hong Kong Chinese population with Crohn’s disease
Source: Gut Microbes. 2021 Nov 23;13(1):1994833. doi: 10.1080/19490976.2021.1994833 (PMC8632309; doi:10.1080/19490976.2021.1994833)
Supplement: Supplemental Material [file KGMI_A_1994833_SM5525.zip › Supplementary legends.docx]

**Supplementary figure 1** (A) Mucosal *E. coli* load in inflamed, non-inflamed and control tissues. (B) Mucosal *E. coli* load in paired inflamed and non-inflamed tissues taken from CD patients with partial inflamed terminal ileum. (C) Invasion rate of *E. coli* isolates from inflamed and non-inflamed tissues. (D) Mucosal *E. coli* load in AIEC-positive and AIEC negative tissues. AIEC load was represented by CFU per milligram ileal tissue after logarithmic transformation. (E) Chao1 Richness and Shannon diversity index of CD tissues and control tissues.

**Supplementary figure 2** Average performance of RF model by sequential forward selection. Best performance was achieved when using 12 features. RF, random forest. Error rate refers to the percentage of misclassified samples at each iteration.

**Supplementary figure 3** Mucosal differentially abundant genera between AIEC-infected mice and K12 mice determined by LEfSe. Taxa with LDA>2 and adjusted p<0.05 were shown. LEfSe: Linear discriminant analysis Effect Size; LDA: Linear discriminant analysis.

**Supplementary figure 4** Faecal AIEC load in mice receiving DSS, with or without antibiotics prior to AIEC administration. In our preliminary experiment (n=4 per group), AIEC 62d was able to colonize the mouse gut when mice were treated with a 7-day course of DSS and persisted in the gut for at least 21 days after initial inoculation, with or without antibiotic treatment prior to gavage (ampicillin 1 g/l, meropenem 500 mg/l and metronidazole 1 g/l). There was no significant difference in faecal AIEC level in mice with or without antibiotic treatment after day 7. AIEC load was represented by CFU per milligram ileal tissue.

**Supplementary table 1.** Clinical characteristics and presence of AIEC in study subjects.

**Supplementary table 2.** Differential pathways between AIEC positive and AIEC negative microbiota.

**Supplementary table 3.** Number of animal samples in each group.
