# Supplementary material for: Association of Adherent-invasive Escherichia coli with severe Gut Mucosal dysbiosis in Hong Kong Chinese population with Crohn’s disease
Source: Gut Microbes. 2021 Nov 23;13(1):1994833. doi: 10.1080/19490976.2021.1994833 (PMC8632309; doi:10.1080/19490976.2021.1994833)
Supplement: Supplemental Material [file KGMI_A_1994833_SM5525.zip › Supplementary table 1.pdf]

Supplementary table 1. Clinical characteristics and presence of AIEC in study subjects

subjectID	condition	paired_samples	AIEC_presence	16s	AIEC_strain	disease
1	inflamed	N	Y	Y	1010a	CD
2	non-inflamed	N	N	Y		CD
3	inflamed	N	N			CD
4	non-inflamed	Y	Y	Y	1099a	CD
4	inflamed	Y	Y	Y	1099a	CD
5	non-inflamed	N	Y	Y	1100a	CD
6	inflamed	N	N	Y		CD
6	non-inflamed	Y	N			CD
7	non-inflamed	N	Y	Y	1105d	CD
8	non-inflamed	N	Y	Y	1111a	CD
9	non-inflamed	N	N			CD
10	non-inflamed	N	N	Y		CD
11	non-inflamed	N	N			CD
12	non-inflamed	N	N			CD
13	non-inflamed	N	N			CD
14	non-inflamed	N	N			CD
15	non-inflamed	N	N			CD
16	inflamed	N	Y	Y	1133a	CD
17	non-inflamed	N	N			CD
18	inflamed	N	N			CD
19	non-inflamed	Y	N			CD
19	inflamed	Y	N			CD
20	non-inflamed	N	N			CD
21	inflamed	N	N	Y		CD
22	non-inflamed	N	N			CD
23	non-inflamed	N	N			CD
24	non-inflamed	Y	Y	Y	1162d	CD
24	inflamed	Y	Y	Y	1162d	CD
25	non-inflamed	N	N	Y		CD
26	non-inflamed	N	Y	Y	1177c	CD
27	non-inflamed	N	N			CD
28	inflamed	N	N			CD
29	inflamed	Y	Y	Y	1186IFc	CD
29	non-inflamed	Y	Y	Y	1186NIFa	CD
30	inflamed	N	N	Y		CD
31	inflamed	N	N	Y		CD
32	non-inflamed	N	Y	Y	1194b	CD
33	inflamed	N	Y	Y	1200c	CD
34	non-inflamed	N	N	Y		CD
35	inflamed	N	Y	Y	1218a	CD
36	non-inflamed	N	Y	Y	1219a	CD
37	non-inflamed	N	Y	Y	1221a	CD
38	non-inflamed	N	Y	Y	1222a&1222b	CD
39	inflamed	N	N	Y		CD
40	inflamed	N	N			CD
41	inflamed	N	N			CD
42	non-inflamed	N	N	Y		CD
43	non-inflamed	N	N	Y		CD

subjectID	condition	paired_samples	AIEC_presence	16s	AIEC_strain	disease
44	inflamed	N	N	Y		CD
45	non-inflamed	N	N	Y		CD
46	non-inflamed	N	N	Y		CD
47	non-inflamed	N	N			CD
48	inflamed	N	N	Y		CD
49	non-inflamed	N	N			CD
50	non-inflamed	N	N			CD
51	non-inflamed	N	Y	Y	1272a	CD
52	non-inflamed	N	Y	Y	1273c	CD
53	non-inflamed	N	N			CD
54	inflamed	N	N	Y		CD
55	non-inflamed	N	Y	Y	1282a	CD
56	non-inflamed	N	N			CD
57	non-inflamed	N	N			CD
58	non-inflamed	N	N			CD
59	non-inflamed	N	N	Y		CD
60	non-inflamed	N	N			CD
61	HC	N	N	Y		HC
62	HC	N	Y	Y	3003b	HC
63	HC	N	N			HC
64	HC	N	Y	Y	3013b	HC
65	HC	N	N	Y		HC
66	HC	N	N	Y		HC
67	HC	N	N	Y		HC
68	HC	N	N	Y		HC
69	HC	N	N	Y		HC
70	HC	N	N	Y		HC
71	HC	N	N	Y		HC
72	HC	N	N	Y		HC
73	HC	N	N			HC
74	HC	N	N			HC
75	HC	N	N	Y		HC
76	HC	N	N	Y		HC
77	HC	N	N	Y		HC
78	HC	N	N	Y		HC
79	HC	N	N	Y		HC
80	HC	N	N			HC
81	HC	N	N	Y		HC
82	HC	N	N	Y		HC
83	HC	N	N			HC
84	HC	N	N			HC
85	HC	N	N			HC
86	HC	N	Y	Y	8223a	HC
87	HC	N	N	Y		HC
88	HC	N	N	Y		HC
89	HC	N	Y	Y	8226a	HC
90	HC	N	N	Y		HC
91	HC	N	N			HC
92	HC	N	N	Y		HC

subjectID	condition	paired_samples	AIEC_presence	16s	AIEC_strain	disease
93	HC	N	N	Y		HC
94	HC	N	N			HC
95	HC	N	N			HC
96	HC	N	N			HC
97	HC	N	N			HC
98	HC	N	N			HC
99	HC	N	N			HC
100	HC	N	N			HC
101	HC	N	N			HC
102	HC	N	N			HC
103	HC	N	N			HC
104	HC	N	N			HC
105	HC	N	N			HC
106	HC	N	N			HC
107	HC	N	N			HC
108	HC	N	N			HC
109	HC	N	N			HC
110	HC	N	N			HC
111	HC	N	N			HC
112	HC	N	N			HC
113	HC	N	N			HC
114	HC	N	N			HC
115	HC	N	N			HC
116	HC	N	N			HC
