# Supplementary material for: Association of Adherent-invasive Escherichia coli with severe Gut Mucosal dysbiosis in Hong Kong Chinese population with Crohn’s disease
Source: Gut Microbes. 2021 Nov 23;13(1):1994833. doi: 10.1080/19490976.2021.1994833 (PMC8632309; doi:10.1080/19490976.2021.1994833)
Supplement: Supplemental Material [file KGMI_A_1994833_SM5525.zip › Supplementary table 2.pdf]

Supplementary table 2. Differential pathways between AIEC positive and AIEC negative microbiota

Pathway	Enriched in	LDA	q_value	parental pathway
VALSYN-PWY: L-valine-Biosynthesis	AIEC negative	2.232	0.044	Amino-Acid-Biosynthesis
GLYCOGENSYNTH-PWY: glycogen-Biosynthesis-I-(from-ADP-D-Glucose)	AIEC negative	2.633	0.038	Carbohydrates-Biosynthesis
ARGSYN-PWY: L-arginine-Biosynthesis-I-(via-L-ornithine)	AIEC negative	2.272	0.015	Amino-Acid-Biosynthesis
PWY-6163: chorismate-Biosynthesis-from-3-	AIEC negative	2.422	0.038	Aromatic-Compounds-Biosynthesis
ARO-PWY: chorismate-Biosynthesis-I	AIEC negative	2.434	0.021	Aromatic-Compounds-Biosynthesis
PWY-5097: L-lysine-Biosynthesis-VI	AIEC negative	2.260	0.048	Amino-Acid-Biosynthesis
TEICHOICACID-PWY: teichoic-acid-(poly-glycerol)-	AIEC negative	2.016	0.027	Cell-Structure-Biosynthesis
PWY-5101: L-isoleucine-Biosynthesis-II	AIEC negative	2.285	0.041	Amino-Acid-Biosynthesis
PWY-5103: L-isoleucine-Biosynthesis-III	AIEC negative	2.225	0.044	Amino-Acid-Biosynthesis
PWY-7400: L-arginine-Biosynthesis-IV-(archaebacteria)	AIEC negative	2.271	0.015	Amino-Acid-Biosynthesis
PWY-3001: superpathway-of-L-isoleucine-Biosynthesis-I	AIEC negative	2.235	0.048	Amino-Acid-Biosynthesis
DAPLYSINESYN-PWY: L-lysine-Biosynthesis-I	AIEC negative	2.166	0.044	Amino-Acid-Biosynthesis
PWY-7242: D-fructuronate-degradation	AIEC negative	2.334	0.029	Secondary-Metabolite-Degradation
PWY4FS-7: phosphatidylglycerol-Biosynthesis-I-(plastidic)	AIEC negative	2.437	0.021	Lipid-Biosynthesis
PWY-5347: superpathway-of-L-methionine-Biosynthesis-(transsulfuration)	AIEC negative	2.293	0.011	Amino-Acid-Biosynthesis
MET-SAM-PWY: superpathway-of-S-adenosyl-L-methionine-Biosynthesis	AIEC negative	2.112	0.048	Super-Pathways
PWY-5505: L-glutamate-and-L-glutamine-Biosynthesis	AIEC negative	2.740	0.009	Amino-Acid-Biosynthesis
GALACT-GLUCUROCAT-PWY: superpathway-of-hexuronide-and-hexuronate-degradation	AIEC negative	2.291	0.010	Super-Pathways
P161-PWY: acetylene-degradation	AIEC negative	2.535	0.044	Secondary-Metabolite-Degradation
PWY-3081: L-lysine biosynthesis V	AIEC negative	2.289	0.048	Amino-Acid-Biosynthesis
PWY-5973: cis-vaccenate-Biosynthesis	AIEC negative	2.343	0.048	Lipid-Biosynthesis
PWY4FS-8: phosphatidylglycerol-Biosynthesis-II-(non-	AIEC negative	2.437	0.021	Lipid-Biosynthesis
COMPLETE-ARO-PWY: superpathway-of-aromatic-amino-acid-Biosynthesis	AIEC negative	2.425	0.027	Amino-Acid-Biosynthesis
ILEUSYN-PWY: L-isoleucine-Biosynthesis-I-(from-threonine)	AIEC negative	2.232	0.044	Amino-Acid-Biosynthesis
GALACTUROCAT-PWY: D-galacturonate-degradation-I	AIEC negative	2.378	0.036	Carbohydrates-Degradation
GLUCUROCAT-PWY: superpathway-of-β-D-glucuronide-and-D-glucuronate-degradation	AIEC negative	2.380	0.004	Secondary-Metabolite-Degradation
GLYCOCAT-PWY: glycogen-degradation-I-(bacterial)	AIEC negative	2.663	0.041	Carbohydrates-Degradation
FUCCAT-PWY: fucose-degradation	AIEC negative	2.060	0.041	Carbohydrates-Degradation
PWY0-1479: tRNA-processing	AIEC positive	2.412	0.044	Generalized-Reactions
PWY-6467: Kdo-transfer-to-lipid-IVA-III-(Chlamydia)	AIEC positive	2.224	0.036	Cell-Structure-Biosynthesis
PWY-5022: 4-aminobutanoate-degradation-V	AIEC positive	2.261	0.038	Fermentation
PWY-6703: preQ0-Biosynthesis	AIEC positive	2.338	0.048	Secondary-Metabolite-Biosynthesis
PWY0-1586: peptidoglycan-maturation-(meso-diaminopimelate-containing)	AIEC positive	2.493	0.041	Cell-Structure-Biosynthesis
PWY-5695: urate-Biosynthesis/inosine-5'-phosphate-degradation	AIEC positive	2.540	0.048	Nucleotide-Degradation
POLYISOPRENSYN-PWY: polyisoprenoid-Biosynthesis-(E.-	AIEC positive	2.125	0.041	Cofactor-Biosynthesis
PWY-7200: superpathway-of-pyrimidine-deoxyribonucleoside-salvage	AIEC positive	2.014	0.029	Nucleotide-Biosynthesis
HISDEG-PWY: L-histidine-degradation-I	AIEC positive	2.472	0.017	Amino-Acid-Degradation
PWY-7539: 6-hydroxymethyl-dihydropterin-diphosphate-Biosynthesis-III-(Chlamydia)	AIEC positive	2.197	0.027	Cofactor-Biosynthesis
PWY-7323: superpathway-of-GDP-mannose-derived-O-antigen-building-blocks-Biosynthesis	AIEC positive	2.365	0.017	Carbohydrates-Biosynthesis
COLANSYN-PWY: colanic-acid-building-blocks-Biosynthesis	AIEC positive	2.326	0.029	Carbohydrates-Biosynthesis
NAGLIPASYN-PWY: lipid-IVA-Biosynthesis	AIEC positive	2.357	0.033	Cell-Structure-Biosynthesis
PWY-1269: CMP-3-deoxy-D-manno-octulosonate-	AIEC positive	2.384	0.025	Carbohydrates-Biosynthesis
SALVADEHYPOX-PWY: adenosine-nucleotides-degradation-	AIEC positive	2.857	0.029	Nucleotide-Degradation
