# Supplementary material for: Association of Adherent-invasive Escherichia coli with severe Gut Mucosal dysbiosis in Hong Kong Chinese population with Crohn’s disease
Source: Gut Microbes. 2021 Nov 23;13(1):1994833. doi: 10.1080/19490976.2021.1994833 (PMC8632309; doi:10.1080/19490976.2021.1994833)
Supplement: Supplemental Material [file KGMI_A_1994833_SM5525.zip › Supplementary table 3.pdf]

Supplementary table 3. Number of animal samples in each group.

Number of mice in each group				
	AIEC	K12	control	
before FMT	6	6		
after FMT	8	8		
after PBS	8	8		
			6	
total	22	22	6	50

Number of mice stool samples sequenced in each group				
	AIEC	K12	control	
before FMT	3	3		
after FMT	6	4		
			1	
total	9	7	1	17

Number of mice tissue samples sequenced in each group				
	AIEC	K12	control	
after FMT	6	4		
			2	
total	6	4	2	12
