# Supplementary figures and images for: Association of Adherent-invasive Escherichia coli with severe Gut Mucosal dysbiosis in Hong Kong Chinese population with Crohn’s disease
Source: Gut Microbes. 2021 Nov 23;13(1):1994833. doi: 10.1080/19490976.2021.1994833 (PMC8632309; doi:10.1080/19490976.2021.1994833)

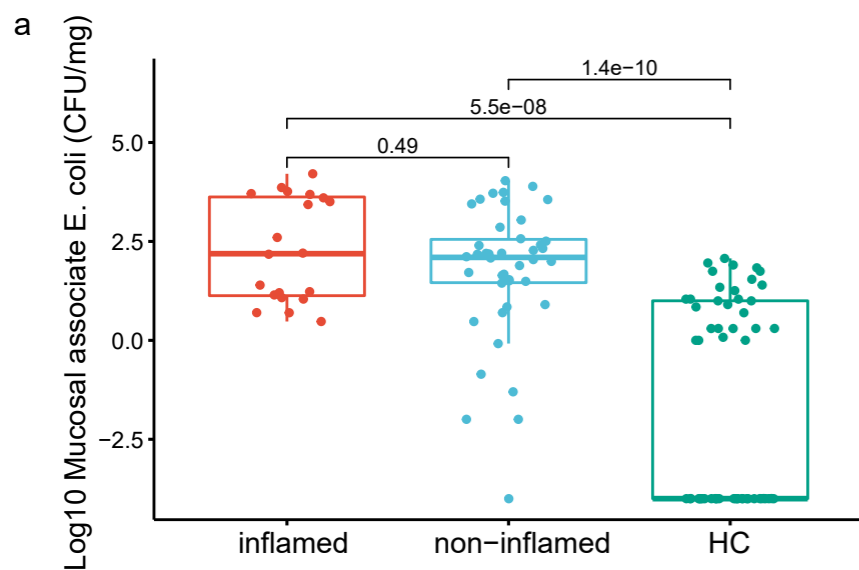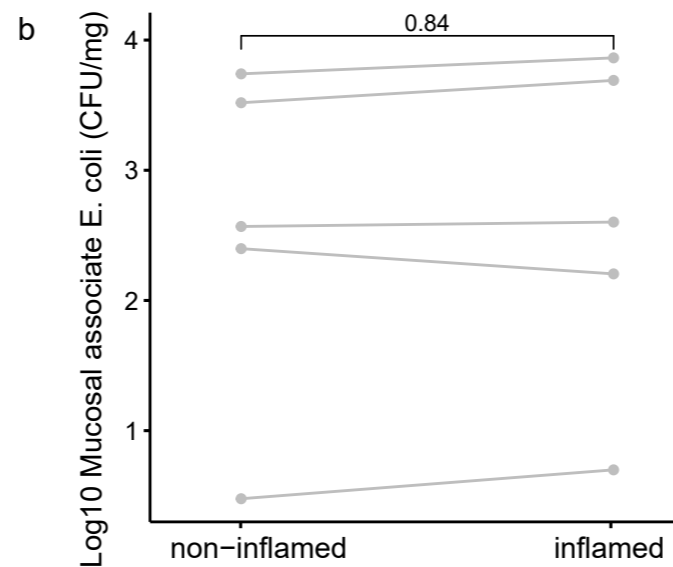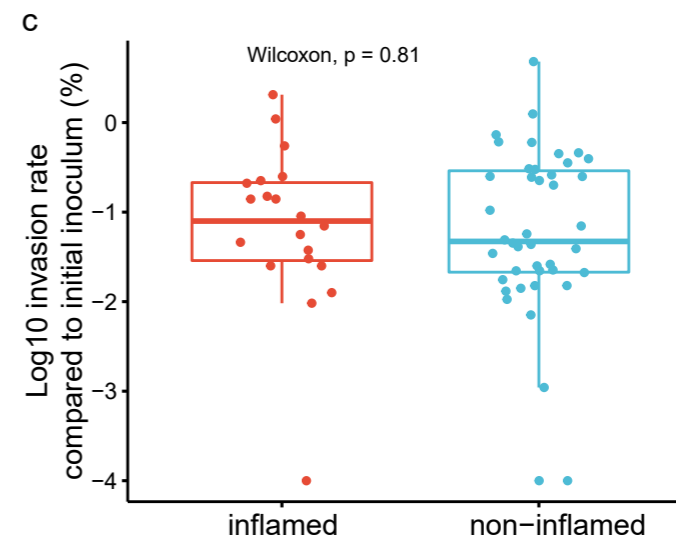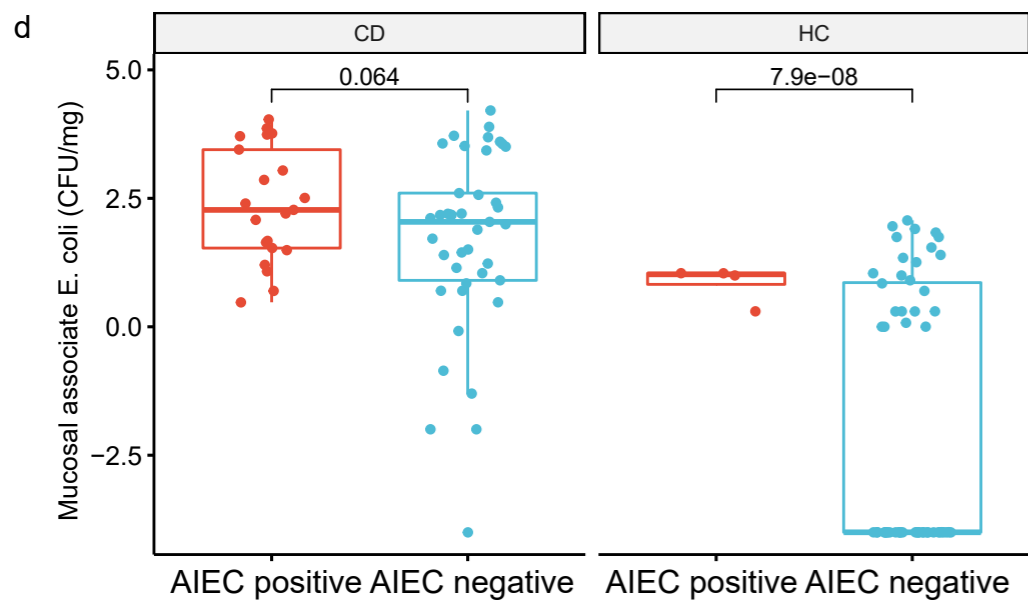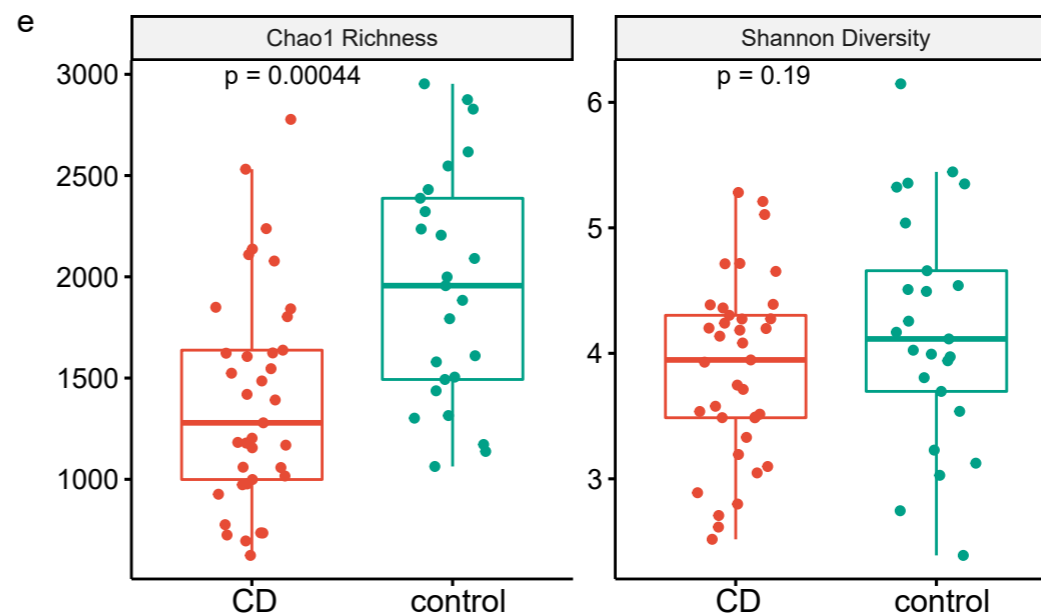

Supplement: Supplemental Material [file KGMI_A_1994833_SM5525.zip › figure S1.pdf]

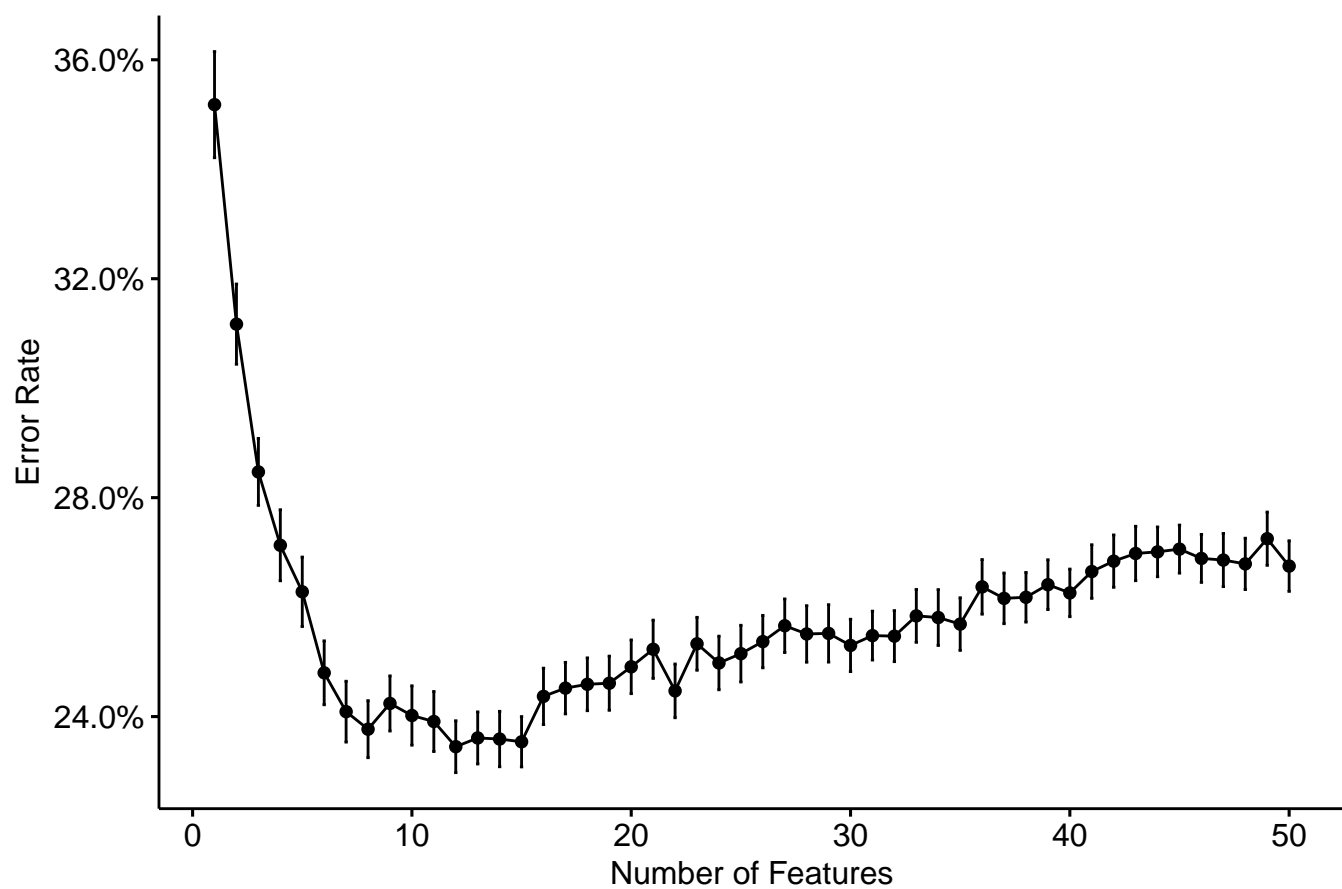

Supplement: Supplemental Material [file KGMI_A_1994833_SM5525.zip › figure S2.pdf]

Enriched in the mucosa of K12 mice after FMT

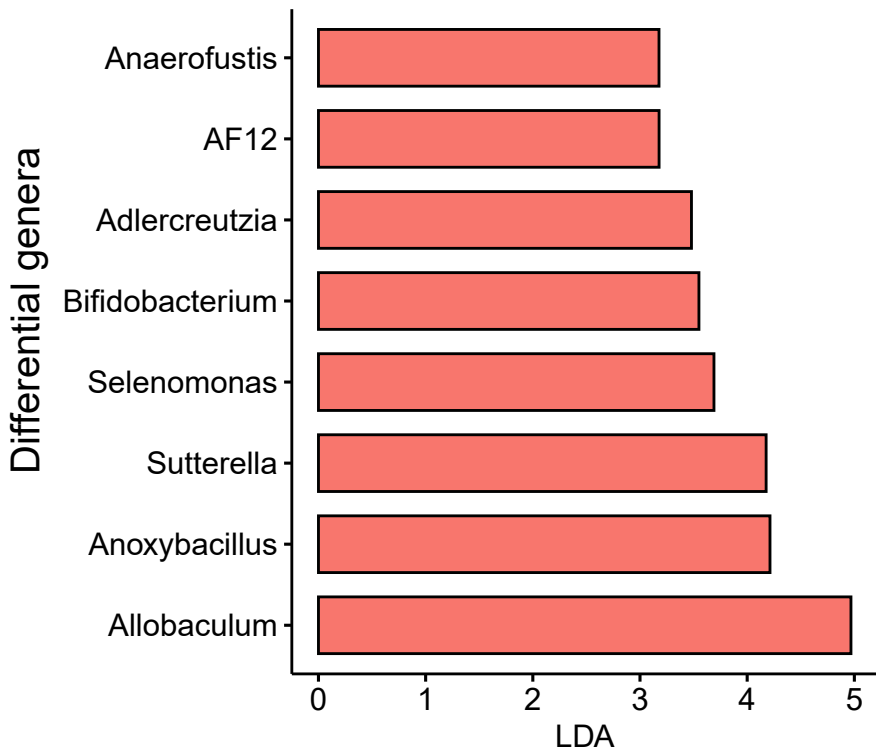

Supplement: Supplemental Material [file KGMI_A_1994833_SM5525.zip › figure S3.pdf]

## AIEC in stool

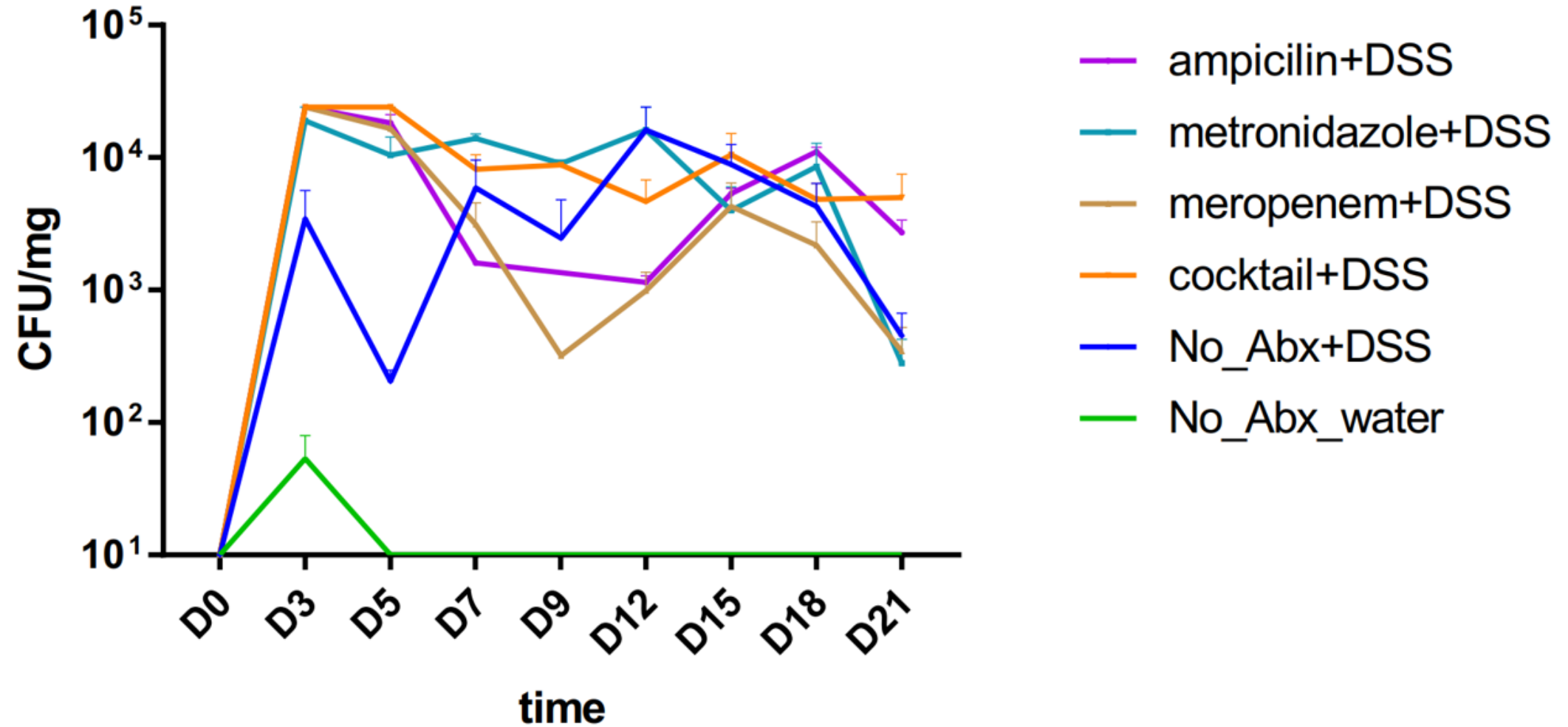

Supplement: Supplemental Material [file KGMI_A_1994833_SM5525.zip › figure S4.pdf]
